# Features and Diagnostic Accuracy of Optical Coherence Tomography Angiography in Neovascular Age-related Macular Degeneration

**DOI:** 10.7759/cureus.6485

**Published:** 2019-12-28

**Authors:** Maria Usman, Kashif Iqbal, Muhammad Hassaan Ali, Khurram Nafees

**Affiliations:** 1 Division of Vitreoretinal Surgery, Layton Rehmatulla Benevolent Trust (LRBT) Free Eye Hospital, Lahore, PAK; 2 Ophthalmology: Vitreo-retina, Layton Rehmatulla Benevolent Trust (LRBT) Free Eye Hospital, Lahore, PAK; 3 Ophthalmology, Allama Iqbal Medical College/Jinnah Hospital, Lahore, PAK; 4 Ophthalmology, FMH College of Medicine and Dentistry, Lahore, PAK

**Keywords:** choroidal neovascularization, fundus fluorescein angiography, neovascular age related macular degeneration, optical coherence tomography angiography

## Abstract

Introduction

Age-related macular degeneration (AMD) is one of the important causes of visual impairment in aging population. Fundus fluorescein angiography (FFA) is gold standard for the diagnosis of neovascular AMD (nAMD) while optical coherence tomography (OCT) aids in the diagnosis of nAMD and is very useful for follow-up. OCT angiography (OCTA) is an evolving imaging technology that can be used as a valid diagnostic tool to study morphology of choroidal neovascularization (CNV) that is seen in nAMD. This study was conducted with the objective to determine diagnostic accuracy and OCTA features of occult and classic CNV in patients with nAMD.

Methods

In this prospective observational study, 90 eyes of 58 patients with nAMD were studied with OCT, OCTA and FFA. OCTA scans were analyzed to qualitatively describe the morphological appearance of CNV in terms of location, pattern and configuration. The OCTA sensitivity and specificity for CNV detection were calculated by comparing it with FFA.

Results

FFA detected CNV in 70 of the 90 eyes (77.77%) whereas OCTA identified CNV in 69 eyes (76.7%). Among 69 eyes with CNV, it was well-defined in 51 (73.9%) eyes and poorly defined in 18 (26.1%) eyes. There were four false positive and five false negative cases. The sensitivity, specificity, positive predictive value and negative predictive value of OCTA in detection of nAMD were found to be 92.85%, 80.0%, 94.2 and 76.2, respectively.

Conclusion

OCTA is a useful, noninvasive, reproducible imaging tool for diagnosing, classifying and localizing CNV. The technique has high sensitivity and specificity and can be used reliably in cases where FFA is contraindicated or inconclusive.

## Introduction

Age-related macular degeneration (AMD) is one of the very important causes of legal blindness in the world in age over 50 years, with a prevalence of 6.8% in Asia. The projected number of people with AMD in 2020 and 2040 is expected to be 196 million and 288 million, respectively [1]. The two types of AMD are non-neovascular or dry AMD and neovascular or wet AMD. The neovascular AMD (nAMD) is responsible for 90% of acute blindness in patients affected with AMD [2].

nAMD manifests as choroidal neovascularization (CNV), pigment epithelial detachment (PED), retinal pigment epithelial tears, disciform scarring, and intraretinal hemorrhages [2]. CNV is the hallmark of nAMD and is characterized by the growth of new vessels originating from the choriocapillaries through a breach in the Bruch’s membrane into the subretinal or sub-retinal pigment epithelial (RPE) space [3]. Type 1 or occult CNV lies beneath RPE while Type 2 or classic CNV lies above or at the level of RPE. Type 3 is retinal angiomatous proliferation (RAP) which results from neovessels originating from the deep retinal capillary plexus and growing downwards to anastomose with choriocapillaris [4].

The clinical assessment of CNV includes visual acuity and dilated fundus examination. Conventional imaging of CNV is done with indocyanine green angiography (ICGA) and fundus fluorescein angiography (FFA) which are invasive and require dye injection. Optical coherence tomography (OCT) angiography (OCTA) is a novel, non-invasive imaging modality which does not require any dye injection and gives high quality images which can be reconstructed in three dimension to see neovascularization on the retina [5].

FFA is useful in evaluating many retinal disorders and is considered as a gold standard for the diagnosis of CNV [6]. The leakage of dye in the late frames of FFA helps the clinician in diagnosing and classifying CNV as classic, occult and their combination that includes minimally classic and predominantly classic. Despite its utility in visualizing vascular leakage, FFA is an invasive, time consuming procedure and is associated with various side effects like nausea, vomiting and anaphylaxis [7].

OCTA is an emerging imaging technology that gives cross-sectional, three-dimensional and high resolution view of the retinal and choroidal vasculature [8]. OCTA compares the decorrelation signal between sequential OCT B-scans taken at precisely the same cross-section in order to create a map of blood flow [9]. It gives clear idea of the size, structure, configuration, and location of the neovessels. It is non-invasive and therefore, has no dye-related adverse reactions. Being visual, informative and retrievable it has many applications in several retinal diseases [10-13]. We conducted this study to determine the diagnostic accuracy of OCTA in diagnosing nAMD, compared with FFA, and to study the distinctive OCTA patterns and features of CNV in nAMD patients.

## Materials and methods

This observational cross-sectional study was conducted at Layton Rehmatulla Benevolent Trust Eye Hospital, Lahore, Pakistan from March, 2018 to July, 2019. The study was conducted after obtaining approval of its synopsis from Ethical Review Board of the same institution and adhered to the principles of ethical medical practice as laid down in Declaration of Helsinki 2011. An informed written consent was obtained from all the patients before enrolling them for the study.

We studied 90 eyes of 58 patients meeting the inclusion criteria which included patients with nAMD, suspicion of CNV at macula with AMD features of drusens and RPE changes, age above 50 years and adequately clear ocular media. Both the treatment-naive eyes and those under anti-VEGF treatment were included. Patients with dry AMD with no signs of CNV, or CNV due to other conditions like angioid streaks, trauma and myopia were excluded from the study. Patients having co-existent diabetic retinopathy, diabetic maculopathy, hypertensive retinopathy, retinal vein occlusion and pathological myopia were also excluded from the study.

All patients underwent comprehensive clinical assessment including visual acuity, slit lamp biomicroscopy, FFA, OCT and OCTA. FFA, OCT and OCTA scans were evaluated by two different researchers (MU and KI) to reduce interobserver bias.

FFA was performed with Topcon TRC 50DX retinal imaging camera. The contrasting technique was standard and CNV was graded according to Macular Photocoagulation Study (MPS) as either occult, classic or combined (predominantly or minimally classic) [12,13]. Classic CNV was defined as a uniform area of hyperfluorescence appearing in early frame (<30 sec) and increasing in late frames. The occult CNV was characterized by fibrovascular PED having hyperfluorescent stippling or late leakage of undetermined source (LLUS).

OCT and OCTA scans were performed on Nidek RS-3000 using 3D Macula Map and Retinal Angio scanning algorithms with in-built eye tracker. In case of impaired fixation, patients were re-scanned until clear images with no motion artifacts were obtained. CNV was identified as hyperreflective fibrovascular complex located below RPE (subRPE/occult) or above RPE (subretinal/classic). The associated RPE disruption, subretinal and intraretinal fluid, neurosensory retina (NSR) surface irregularity, PEDs and drusens were also noted.

For OCTA, macular zone was imaged using an area size measuring 3 × 3 mm and 6 × 6 mm (in cases of larger vascular network). The enface images of OCTA were analyzed after automatic segmentation into four layer: choroid, outer retina, deep vascular plexus, superficial vascular plexus. In majority of the patients, the base layer was selected as RPE/basement membrane (BM) curve in order to enhance the image quality. OCTA scan was labelled positive in the presence of abnormal flow signal in the choroid and/or the outer retina enface images. The location, pattern, configuration, and extent of visualization of CNV network were noted.

OCTA findings were compared with FFA for characterizing CNV lesions. Specificity, sensitivity, positive and negative predictive values (PPV and NPV) were calculated to determine diagnostic accuracy of OCTA taking FFA as gold standard. An eye having CNV on both FFA and OCTA was labelled as true positive whereas an eye with CNV on FFA alone but not detected on OCTA was labelled as false negative. Similarly, if FFA showed no CNV but OCTA revealed lesion, it was labelled as false positive whereas an eye showing no lesion on either FFA or OCTA was labelled as true negative.

Statistical analysis was performed using Statistical Package for Social Sciences (SPSS, IBM Statistics, Chicago, IL, USA version 23.0). Mean ± SD was calculated for numerical variables whereas frequencies and percentages were calculated for descriptive variables.

## Results

On examination, 90 eyes of 58 consecutive patients met the inclusion criteria. Among the 90 eyes, 65 eyes had clear signs of neovascular ARMD and 25 eyes had suspicion of CNV. The mean age of the study population was 58.5 ± 5.05 years. CNV was bilateral in 32 patients (64 eyes) and unilateral in 26 patients.

**Figure 1:**
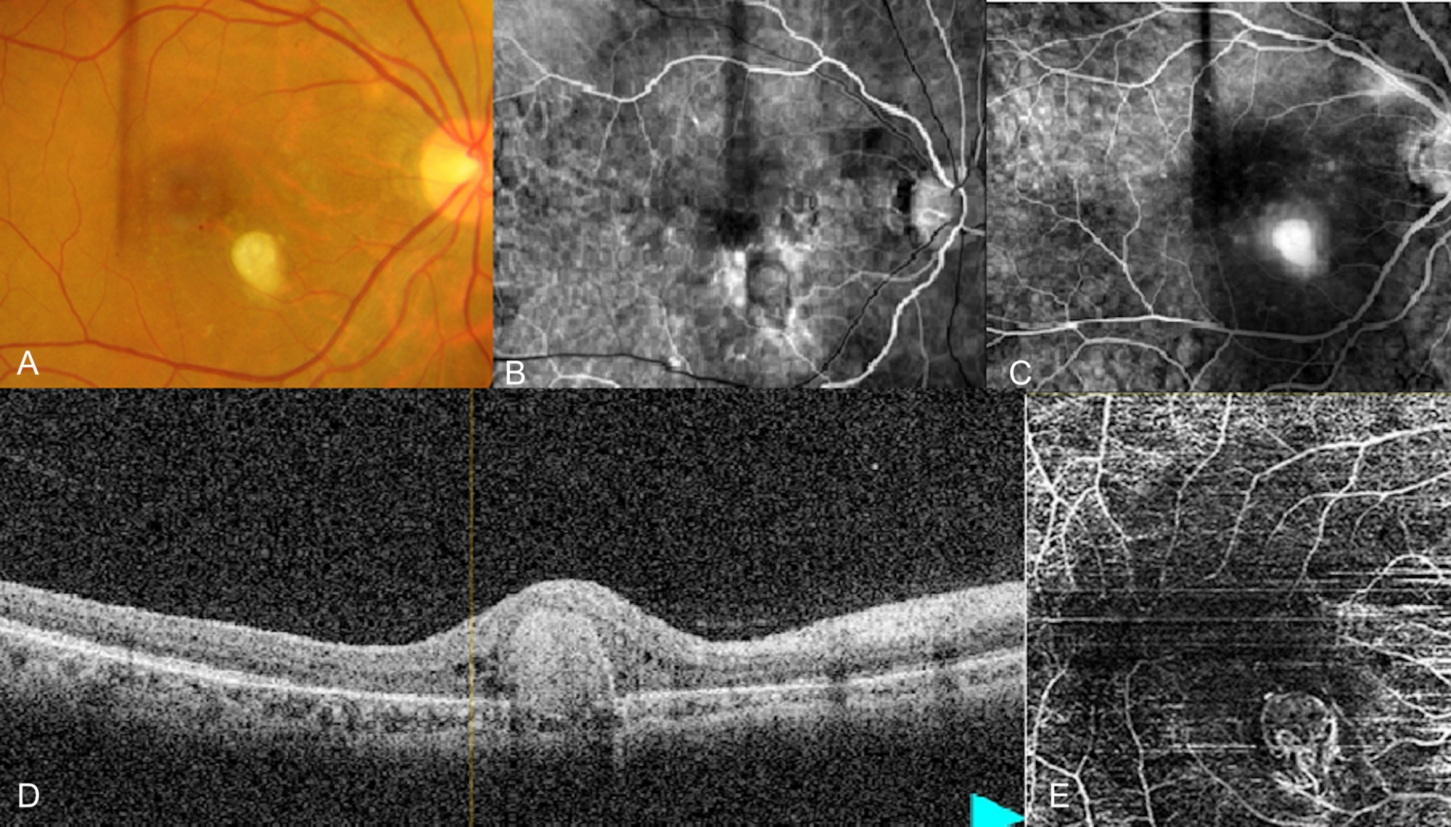
Features of classic CNV on FFA and OCTA (A) Clinical photograph showing yellow colored lesion in the macular area inferior nasal to fovea. (B) Early frame of FFA showing vascularization in the foveal area which is usually avascular. (C) Late frame of FFA showing remnant staining at the foveal area. (D) OCT image shows breakdown of the retinal pigment epithelium possibly due to underlying vascular proliferation. (E) OCTA image shows increased vascular bed in the macular area with central avascular area in the center corresponding to fovea. CNV: Choroidal neovascularization; FFA: Fundus fluorescein angiography; OCT: Optical coherence tomography; OCTA: Optical coherence tomography angiography.

FFA revealed CNV in 70 of the 90 eyes (77.8%) whereas 20 (22.2%) eyes did not show hyperfluorescence in any frame, indicating no CNV. They were considered as controls. On FFA, 38 (54.3%) eyes had classic, 21 (30.0%) eyes had occult and 11 (15.7%) eyes had combined CNVs. Six (8.6%) eyes had predominantly classic whereas five (7.1%) eyes had minimally classic lesions (Figure 1).

**Figure 2:**
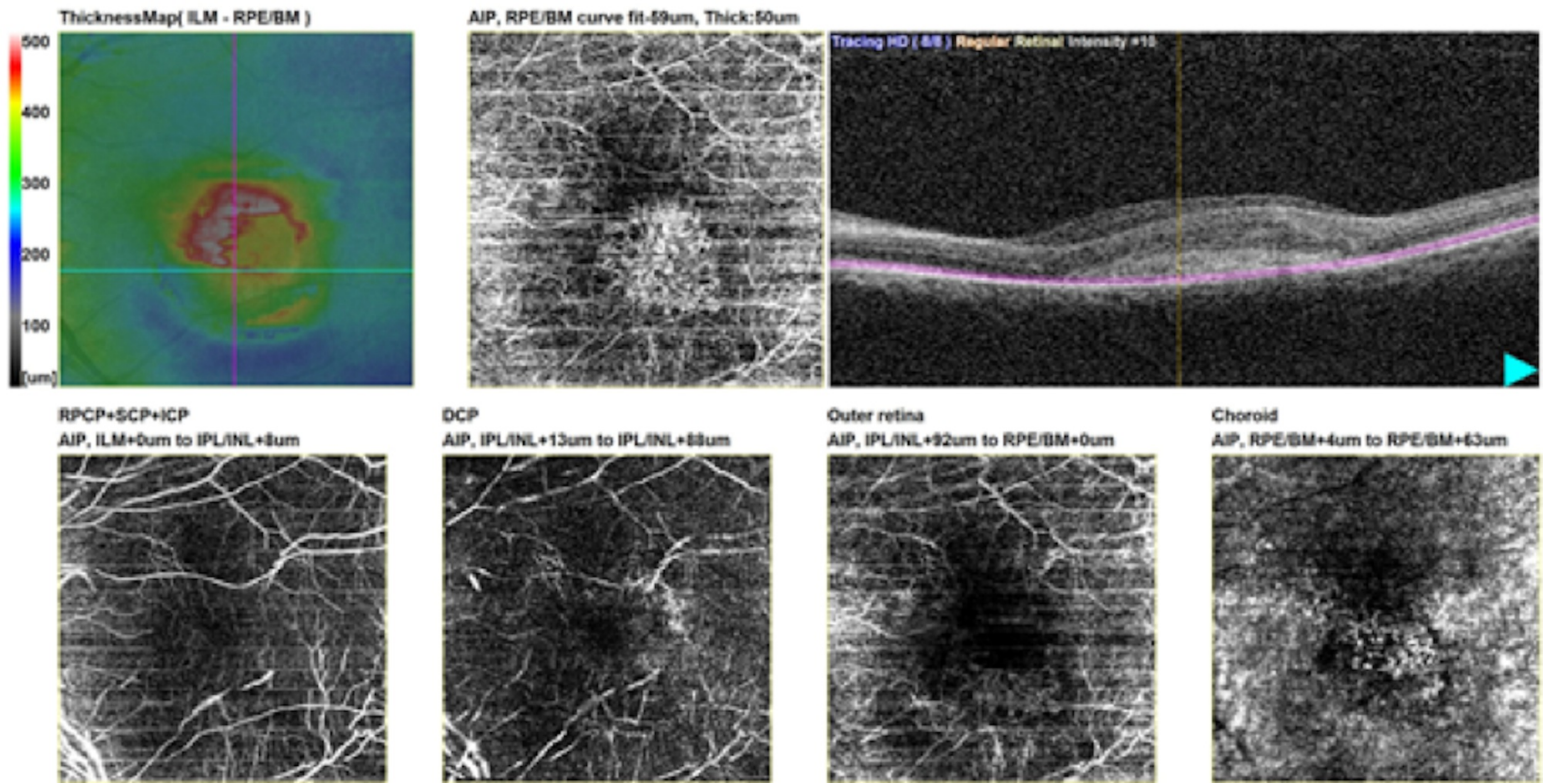
Enface image of occult/sub-RPE CNV having ill-defined pattern RPE: Retinal pigment epithelium; CNV: Choroidal neovascularization.

The qualitative analysis of OCTA showed choroidal neovascular network in 69 eyes (76.7%). Sub-RPE complexes originating from choriocapillaris and not seen in outer retina window of enface images, indicating occult CNVs, were seen in 24 (34.8%) eyes (Figure 2).

**Figure 3:**
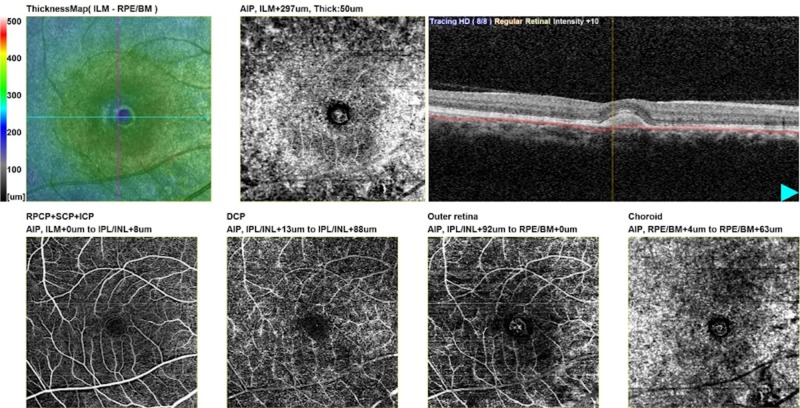
Enface OCTA images of fibrovascular PED OCTA: Optical coherence tomography angiography; PED: Pigment epithelial detachment.

Subretinal vascular complexes seen in the outer retina and choroidal windows of enface images, showing classic CNVs, were seen in 45 (65.2%) eyes. Of these, 40 (57.9%) eyes had their major neovascular complexes lying in the outer retinal window and were originating from choriocapillaris in the choroid window. Drusens and serous PEDs with no occult CNV were seen in 16 eyes that were labelled as true negatives (Figure 3).

**Figure 4:**
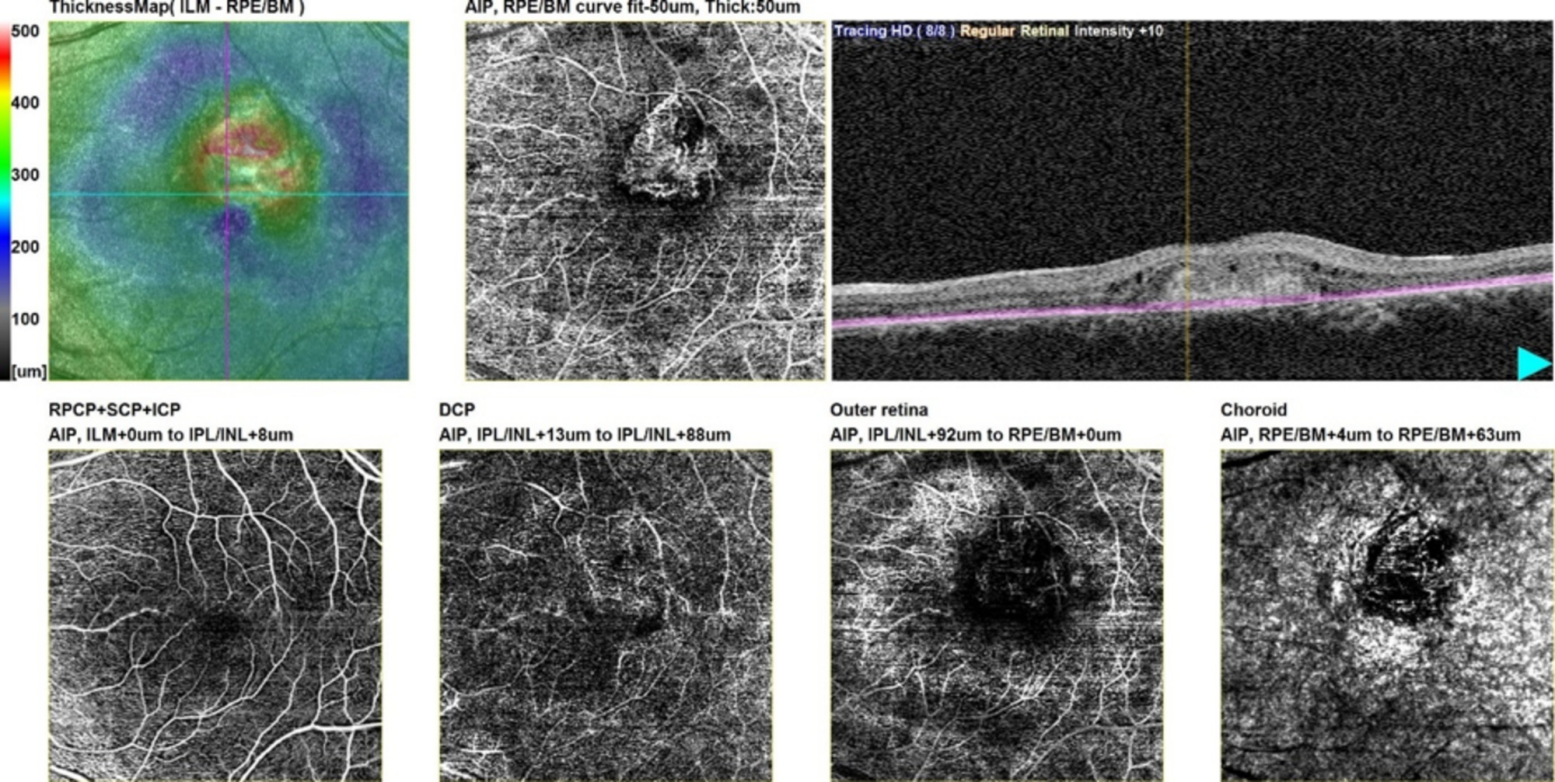
Type 3 choroidal neovascularization: retinal angiomatous proliferation Feeder vessel of neovascularization seen originating from deep retinal plexus.

As demonstrated in Table 1, there were four false positive eyes. Their scans showed small loop-like neovascularization in the choroid window, not picked by FFA. Two of these eyes were under anti-VEGF treatment and were found to have residual CNV. In the other two eyes, OCTA also picked early CNVs; these were in patients with classic CNVs in fellow eyes. There were five false negative eyes of which one had extensive subretinal hemorrhage at macula obscuring the signal passage through RPE. One eye had dense exudation adjacent to CNV causing extreme distortion of RPE that could not be overcome by manual adjustment. Retinal angiomatous proliferation (RAP) type 3 CNV was identified in three eyes, shown by anastomotic link between inner and outer retinal plexuses with no disruption of RPE (Figure 4).

As illustrated below, among the sub-RPE/occult CNV 79.16% eyes (18/24) had loop-like configuration. Well-defined pattern was seen in 58.3% eyes (14/24) while 41.7% eyes (10/24) had ill-defined pattern. Among the subretinal/classic CNVs, 82.2% (37/44) eyes had well defined pattern of neovascular tufts, 51.1% (23/45) eyes had tree-like while 48.9% (22/45) eyes had loop-like configuration (Table 1).

**Table 1:** Neovascular complexes in eyes with classic and occult CNV as detected on OCTA CNV: Choroidal neovascularization; OCTA: Optical coherence tomography angiography.

Type of CNV	Features of CNV				Total n (%)
	Boundaries		Configuration		
	Well-defined n (%)	Ill-defined n (%)	Loop-like n (%)	Tree-like n (%)	
Classic	37 (82.2)	8 (17.8)	22 (48.9)	23 (51.1)	45 (65.2)
Occult	14 (58.3)	10 (41.7)	18 (75.0)	6 (25.0)	24 (34.8)
Total	51 (73.9)	18 (26.1)	40 (58.0)	29 (42.0)	69 (100.0)

The sensitivity, specificity, positive predictive value and negative predictive value were found to be 92.85%, 80.0%, 94.2 and 76.2, respectively (Table 2).

**Table 2:** 2×2 contingency table computing relative CNV between OCTA and FFA CNV: Choroidal neovascularization; FFA: Fundus fluorescein angiography; OCTA: Optical coherence tomography angiography.

OCTA	FFA		Total
	Positive	Negative	
Positive	65	4	69
Negative	5	16	21
Total	70	20	90

One eye under anti-VEGF treatment had residual CNV on OCTA but not on FFA. The area of CNV in enface image reduced with anti-VEGF therapy in nine eyes followed up on OCTA monthly for 3-4 months (average 3.5 months). It was also observed that the area of CNV in enface image reduced with anti-VEGF therapy in nine eyes followed up on OCTA monthly for 3-4 months (average 3.5 months).

## Discussion

Conventional FFA is widely used to study retinal and choroidal vasculopathies but has limitations due to invasiveness and dye-related side effects notably nausea, vomiting and allergic reactions. OCT, on the other hand, is fast and noninvasive, and has emerged as an alternative to FFA to detect and monitor CNV. However, OCT cannot replace traditional angiography as hyperreflectivity of RPE, hemorrhage and drusens have similar signals on OCT and FFA distinguishes between these lesions reasonably well [14].

OCTA detects blood flow in vessel lumen by determining the variation in the reflected OCT signal amplitude among consecutive cross-sectional B-scans [15,16]. It incorporates different algorithms based on variation in amplitude, intensity or phase. The amplitude or intensity-based algorithms are speckle variance, intensity-based optical microangiography (OMAG), intensity-based Doppler variance, split-spectrum amplitude decorrelation angiography (SSADA) and cross-correlation mapping [16]. These methods produce less motion artifacts while phase and complex algorithms, like Doppler variance, phase variance, and OMAG need removal of motion artifacts [17]. OCTA can detect abnormal vasculature in retina and choroid as well as absence of blood flow at macula. It is found to be useful in CNV, diabetic maculopathy, vein occlusions, glaucoma, optic disc disorders and serous retinal detachments besides many other retinal proliferative and anterior segment ischemic disorders [18,19].

According to the study conducted by Gong et al., 56 of 86 eyes had CNV on OCTA. Among 86 eyes, 46.4% (26/56) had well-circumscribed vessels while 53.6% (30/56) revealed poorly circumscribed vessels [11]. The study done by Nikolopoulou et al. reported that OCTA had sensitivity of 88% and specificity of 90% [20]. Another study reported sensitivity and specificity of OCTA to be 89.2% and 93.3%, respectively [13]. The researchers observed that in the occult CNV group, 76.74% eyes had loop-like membranes. The lesions were well-defined in 55.8% of cases and ill-defined in 44.19%. In the classic group, CNV was well-defined in 82.76%, loop-like in 51.72% and tree-like in 42.28% [13]. Carlo et al. calculated the specificity and sensitivity of OCTA in diagnosing nAMD as 50% and 91%, respectively [21].

Our findings clearly demonstrate that OCTA detects neovascular complexes in most of the cases of nAMD, enabling the morphological analysis of the CNV in each and every patient. In intergroup analysis we observed that classic CNV was mostly well-defined tree-like membrane and occult CNV was mostly loop-like well-defined lesion. A feeder trunk originating from choriocapillaris was almost always seen. These results are in concordance to other studies [13,22,23]. It was observed that enface images can also determine the activity of CNV by the density of neovascular patterns seen [24]. The denser the vascular network of CNV on enface image, the more likely it would affect vision and therefore take more time to respond to treatment.

Interestingly, CNV was detected in four eyes on OCTA despite no finding on FFA. Three eyes had evolving occult CNV on OCTA. Two of these eyes were symptomatic and were therefore treated with anti-VEGF. If picked late on FFA, the CNV would have progressed and visual deterioration would have been unavoidable. This shows that OCTA can pick early lesions in some circumstances even when FFA fails to reveal them. One eye under anti-VEGF treatment had residual CNV on OCTA but not on FFA. We also found that the area of CNV in enface image reduced with anti-VEGF therapy in nine eyes followed up on OCTA monthly for 3-4 months (average 3.5 months). The response to anti-VEGF has been observed in other studies [13,25-27]. OCTA also identified four eyes with classic CNV on FFA as RAP type 3, observed as a small, round, well-defined hyperreflective microvascular tuft at the outer capillary plexus level [28]. Therefore, we propose that OCTA can better detect vascular pathologies and give clear diagnosis.

Although informative, this study has a few limitations notably image artifacts. The flow projection artifact created by fluctuating shadows from superficial retinal plexus circulation intricates the interpretation of enface images of deeper vascular plexus [29]. The software processing can remove this artifact but the confluent choriocapillaris mask the view of deep choroid. Adequate knowledge of the potential artifacts and critical analysis of OCTA data helps better interpretation [30]. Secondly, we have used a heterogeneous cohort and future studies are recommended to report data separately in both treatment-naïve and treated patients.

## Conclusions

OCTA clearly identifies occult and classic CNV in nAMD patients by localizing the vascular network. It gives details of network configuration, pattern and vascular density of occult and classic CNV. High sensitivity and specificity advocates excellent plausibility of OCTA results. The valuable information provided by OCTA validates its suitability for monitoring nAMD patients undergoing anti-VEGF therapy. Being able to perform on same machine as OCT, OCTA may replace or at least work adjuvantly to FFA in nAMD management in future.
